# Biologically Active Metabolites Synthesized by Microalgae

**DOI:** 10.1155/2015/835761

**Published:** 2015-08-03

**Authors:** Michele Greque de Morais, Bruna da Silva Vaz, Etiele Greque de Morais, Jorge Alberto Vieira Costa

**Affiliations:** ^1^Laboratory of Microbiology and Biochemistry, College of Chemistry and Food Engineering, Federal University of Rio Grande, P.O. Box 474, 96203-900 Rio Grande, RS, Brazil; ^2^Laboratory of Biochemical Engineering, College of Chemistry and Food Engineering, Federal University of Rio Grande, P.O. Box 474, 96203-900 Rio Grande, RS, Brazil

## Abstract

Microalgae are microorganisms that have different morphological, physiological, and genetic traits that confer the ability to produce different biologically active metabolites. Microalgal biotechnology has become a subject of study for various fields, due to the varied bioproducts that can be obtained from these microorganisms. When microalgal cultivation processes are better understood, microalgae can become an environmentally friendly and economically viable source of compounds of interest, because production can be optimized in a controlled culture. The bioactive compounds derived from microalgae have anti-inflammatory, antimicrobial, and antioxidant activities, among others. Furthermore, these microorganisms have the ability to promote health and reduce the risk of the development of degenerative diseases. In this context, the aim of this review is to discuss bioactive metabolites produced by microalgae for possible applications in the life sciences.

## 1. Introduction

Microalgae are unicellular microorganisms that grow in fresh or salt water and have varied shapes with a diameter or length of approximately 3–10 *μ*m. The term microalgae includes prokaryotic and eukaryotic organisms [1]. Cyanobacteria and bacteria have very similar structural characteristics; however, they are classified as microalgae because they contain chlorophyll *a* and compounds related to photosynthesis. The so-called green algae are so named because of the presence of chlorophyll *a* and chlorophyll *b* in the same proportions as in higher plants [2].

Microalgae are photosynthetic organisms that play a key role in aquatic ecosystems. Approximately 40% of global photosynthesis is due to these microorganisms [3]. Microalgal metabolism reacts to changes in the external environment with changes in its intracellular environment. Thus, the manipulation of the culture conditions, or the presence or absence of certain nutrients, stimulates the biosynthesis of specific compounds.

Several studies have been conducted to investigate the products of microalgal metabolism not only to understand its nature but also to search for substances with possible applications to humans in different fields of interest. Screening of extracts or isolation of metabolites from different microalgae is a common method for determining the biological activity of these components. Microalgae have been described as rich sources of various biocompounds of commercial interest [4].

Bioactive compounds of microalgal origin can be sourced directly from primary metabolism, such as proteins, fatty acids, vitamins, and pigments, or can be synthesized from secondary metabolism. Such compounds can present antifungal, antiviral, antialgal, antienzymatic, or antibiotic actions [5]. Many of these compounds (cyanovirin, oleic acid, linolenic acid, palmitoleic acid, vitamin E, B12, *β*-carotene, phycocyanin, lutein, and zeaxanthin) have antimicrobial antioxidant, and anti-inflammatory capacities, with the potential for the reduction and prevention of diseases [6–9]. In most microalgae, the bioactive compounds are accumulated in the biomass; however, in some cases, these metabolites are excreted into the medium; these are known as exometabolites.

Bioactive metabolites of microalgal origin are of special interest in the development of new products for medical, pharmaceutical, cosmetic, and food industries. Further research should be conducted with these bioactive compounds to verify their beneficial effects for humans, their degradability when released into the environment, and their effects when used in animals [4]. In this context, the aim of this review is to discuss bioactive metabolites produced by microalgae for possible applications in the life sciences.

## 2. Microalgae with Potential for Obtaining Bioactive Compounds

Microalgae are a group of heterogeneous microorganisms that have a great biodiversity of colors, shapes, and cell characteristics, and their manipulation is encompassed by the field of marine biotechnology. Among the thousands of species of microalgae believed to exist, only a small number of them are retained in collections around the world, and it is estimated that only a few hundreds are investigated for compounds present in their biomass. Of these, only and a few are industrially cultivated [9]. This untapped diversity results in potential applications for these microorganisms in several biotechnological fields, such as the production of biocompounds used in food, medicine, cosmetics, and pharmaceuticals and even in the energy industry [10].

Microalgae are a natural source of highly interesting biologically active compounds. These compounds have received much attention from researchers and companies in recent years due to their potential applications in different life science fields. The applications range from the production of biomass for food and feed to the production of bioactive compounds for the medical and pharmaceutical industries [9]. Considering the enormous biodiversity of microalgae and recent developments in genetic engineering, this group of microorganisms is one of the most promising sources for new products and applications [7].

Microalgae are autotrophic microorganisms that use light energy and inorganic nutrients (carbon dioxide, nitrogen, phosphorus, etc.) to develop and synthesize biocompounds that have high aggregated nutritional value and therapeutic functions, such as lipids, proteins, carbohydrates, pigments, and polymers. Recent studies have reported that microalgae can produce different chemical compounds with different biological activities, such as carotenoids, phycobilins, polyunsaturated fatty acids, proteins, polysaccharides, vitamins, and sterols among other chemicals [8, 11, 12].

Components of microalgal origin with antimicrobial, antiviral, anticoagulant antienzymatic, antioxidant, antifungal, anti-inflammatory, and anticancer activity, among others, were identified [13–18]. The study of the extraction of bioactive compounds from various microalgae, such as* Arthrospira (Spirulina), Botryococcus braunii, Chlorella vulgaris, Dunaliella salina, Haematococcus pluvialis, and Nostoc* (Table 1), has been investigated [12, 19, 20].

### 2.1. *Spirulina*

**Figure 1:**
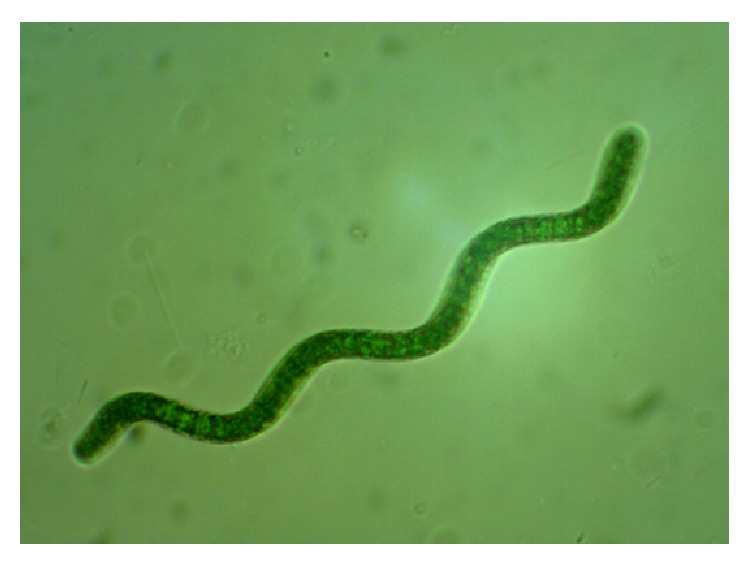
*Spirulina* sp. LEB 18 from LEB/FURG strains bank.

*Spirulina* (*Arthrospira*) is prokaryotic cyanobacteria (Figure 1) that belongs to Cyanophyta, which arose more than 3 million years ago, forming the current oxygen atmosphere, and has been important in the regulation of the planetary biosphere [21]. In 1981,* Spirulina* was approved by the FDA (Food Drug Administration) by the issuance of a GRAS (generally recognized as safe) certificate. The FDA has stated that* Spirulina* can be legally marketed as a food or food supplement without risk to human health [22].

*Spirulina* has a high protein value and high digestibility and contains significant amounts of essential polyunsaturated fatty acids and phenolic compounds [23]. Due to properties such as its high nutritional value and the presence of active biocompounds, this microorganism is one of the most studied microalgae worldwide [24]. The* Spirulina* protein content ranges from 50 to 70% (w/w) of its dry weight, the carbohydrate content from 10 to 20% (w/w), and the lipid content from 5 to 10% (w/w).

This microalga is rich in vitamins B1, B2, B12, and E (especially vitamin B12). Furthermore,* Spirulina* has a high content of pigments, minerals, and oligoelements (approximately 6 to 9% (w/w) biomass dry weight), of which the most important are iron, calcium, magnesium, phosphorus, and potassium [22]. Some studies have demonstrated the use of this microalga for the production of pigments due to its antioxidant properties [25–27]. *β*-Carotene represents approximately 80% of the carotenoids present in* Spirulina,* and other components, such as tocopherols, phycocyanin, and phycoerythrin, are also part of its composition [13].  Table 2 shows some of the bioactive compounds that have been extracted from* Spirulina*.

Cyanobacteria are known to produce intracellular and extracellular metabolites with potential biological activities, such as antibacterial, antifungal, antiviral, antitumor, anti-HIV, anti-inflammatory, antioxidant, antimalarial, herbicidal, and immunosuppressant effects [13, 28, 29]. The therapeutic importance of* Spirulina* has been reported in several studies. These include its use in the treatment of hyperlipidemia, cancer, HIV, diabetes, obesity, and hypertension, the improvement of immune response in renal protection against heavy metals and drugs, and the reduction in serum levels of glucose and lipids, among others [23, 27, 30, 31].

The world's largest producer, Hainan Simai Pharmacy Co. (China), annually produces 3000 tonnes of* Spirulina *biomass [13]. One of the largest industries in the world is Earthrise Farms (California, USA) (http://www.earthrise.com/). Many other companies market a wide variety of nutraceutical products produced from these microalgae. For example, the Myanmar* Spirulina* Factory (Yangon, Myanmar) produces pills, French fries, and pasta. Cyanotech (Hawaii, USA) produces and markets products under the name Spirulina Pacifica (http://www.cyanotech.com/). In Brazil, the Olson Microalgas Macronutrição company (Camaquã, Rio Grande do Sul) produces* Spirulina* sp. LEB 18 capsules for sale as a dietary supplement (http://www.olson.com.br/).

### 2.2. *Nostoc*

**Figure 2:**
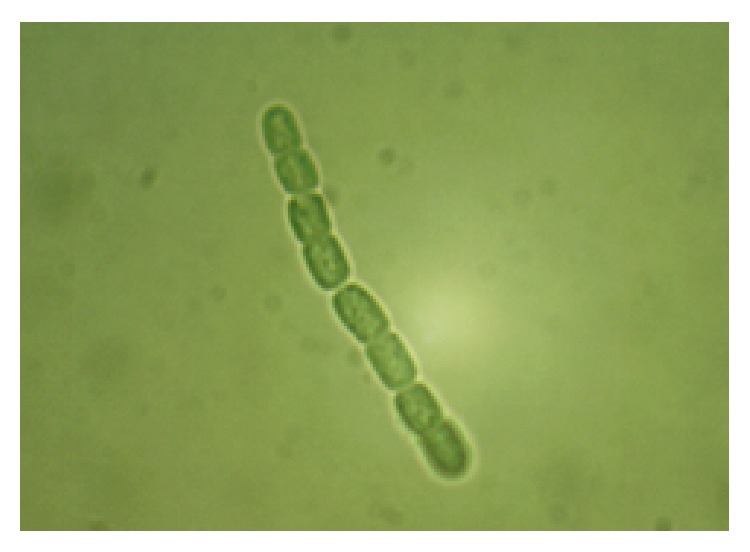
Microalga* Nostoc ellipsosporum* from LEB/FURG strains bank.

*Nostoc* is an edible microalga that belongs to the Nostocaceae group Cyanophyta that forms spherical colonies that link together as filaments. This microalga has heterocysts with a pattern of homogeneous cells and a regular distance between cells that compose the filament (Figure 2) [32]. The heterocysts fix atmospheric nitrogen for amino acid synthesis in the microalgal biomass. In the absence of a nitrogen source during microalgal cultivation, heterocysts form, avoiding the limitation of this nutrient for cell growth [33].

*Nostoc* microalgal biomass has been used in the medical field and as a dietary supplement because of its protein, vitamin, and fatty acid content. The medical value of this microalga was evidenced by its use in the treatment of fistula and for some forms of cancer [34]. Historically, the biomass of this microorganism is described as anti-inflammatory, and it also aids in digestion, blood pressure control, and immune boosting. Several studies suggest that* Nostoc* produces several compounds with antimicrobial, antiviral, and anticancer activity. These results have encouraged its cultivation on a large scale, and it has great economic potential due to its nutritional and pharmaceutical importance [35].  Table 3 presents some bioactive compounds that have been extracted from the microalga of the* Nostoc* genus.

Cyanovirin, a potential protein molecule produced by a* Nostoc* microalga, showed a positive effect in the treatment of HIV [36] and Influenza A (H1N1) [6].* Nostoc* contains a spectrum of polyunsaturated fatty acids (PUFAs) that include essential fatty acids, such as linoleic, *α*-linolenic, *γ*-linolenic, octadecatetraenoic, and eicosapentaenoic acid [37]. Essential fatty acids are precursors of prostaglandins, engendering significant interest from the pharmaceutical industry.

### 2.3. *Chlorella*

**Figure 3:**
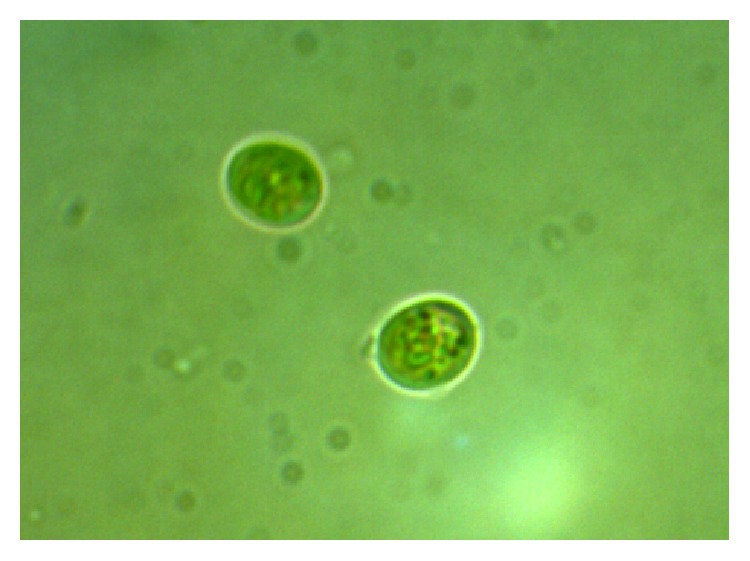
Microalga* Chlorella fusca* LEB 111 from LEB/FURG strains bank.

*Spirulina* and* Chlorella* represent the majority of the microalgal biomass market, with an annual production of 3,000 and 4,000 tons, respectively [38].* Chlorella* sp. is a eukaryotic genus of green unicellular microalgae that belongs to the Chlorophyta group (Figure 3) [39].

This microalga was discovered by the Japanese, traditional consumers of algae, who usually enjoy it and use it as a food supplement. The microalga* Chlorella* is rich in chlorophyll, proteins, polysaccharides, vitamins, minerals, and essential amino acids. This microalga is 53% (w/w) protein, 23% (w/w) carbohydrate, 9% (w/w) lipids, and 5% (w/w) minerals and oligoelements [22].

These nutrient concentrations can be varied by manipulation of culture conditions. The biomass of this microalga is also rich in B complex vitamins, especially B12, which are vital in the formation and regeneration of blood cells. Like* Spirulina*,* Chlorella* has a GRAS certificate issued by the FDA and can thus be used as a food without risk to human health when grown in a suitable environment with proper hygiene and good manufacturing practices [22, 40].

*Chlorella* contains bioactive substances with medicinal properties. Experimental studies with* Chlorella* demonstrated their antitumor, anticoagulant, antibacterial, antioxidant, and antihiperlipidemia effects in addition to a hepatoprotective property and the immunostimulatory activity of enzymatic protein hydrolyzate [39, 41–44].

Many antioxidant compounds may be responsible for* Chlorella* functional activities. Antioxidants such as lutein, *α*-carotene, *β*-carotene, ascorbic acid, and *α*-tocopherol, which are active against free radicals, were identified. Some of these compounds not only are important as natural colorants or additives but also may be useful in reducing the incidence of cancer and in the prevention of macular degeneration [39, 45] (Table 4).

The most important bioactive compound in* Chlorella* is *β*-1,3 glucan, an active immunostimulator that reduces free radicals and blood cholesterol. The efficacy of this compound against gastric ulcers, sores, and constipation has been reported. It also has been demonstrated to have preventive action against atherosclerosis and hypercholesterolemia, as well as antitumor activity [46].* Chlorella* is produced by more than 70 companies. Taiwan Chlorella Manufacturing Co. (Taipei, Taiwan) is the world's largest producer of* Chlorella*, with over 400,000 tons of biomass produced per year (http://www.taiwanchlorella.com/index.php). Significant production also occurs in Klötze (Germany) (80–100 t yr^−1^ of dry biomass) [47].

### 2.4. *Dunaliella*

**Figure 4:**
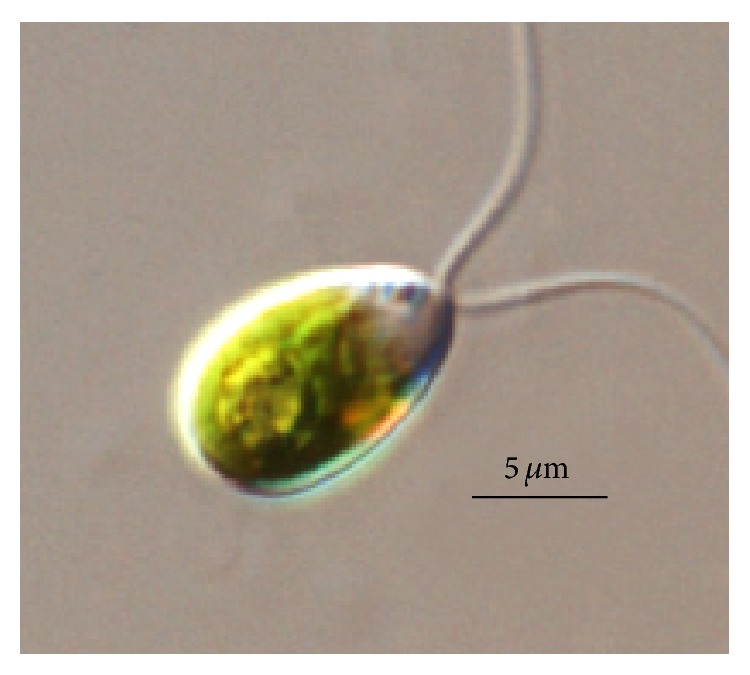
*Dunaliella* sp. microalga [48].

*Dunaliella* is a green unicellular halotolerant microalga that belongs to the Chlorophyceae group (Figure 4). This microalga is widely studied due to its tolerance of extreme habitat conditions, physiological aspects, and its many biotechnological applications.* Dunaliella* is a source of carotenoids, glycerol, lipids, and other bioactive compounds, such as enzymes and vitamins [48, 49].

This microalga is a major source of natural *β*-carotene, able to produce up to 14% of its dry weight under conditions of high salinity, light, and temperature as well as nutrient limitation [50]. In addition to *β*-carotene, this microalga is rich in protein and essential fatty acids, which can be consumed safely, as evidenced by GRAS recognition [22].  Table 5 presents some compounds that have been extracted from microalgae of the* Dunaliella* genus.

Compounds in the* Dunaliella* biomass have various biological activities, such as antioxidant, antihypertensive, bronchodilatory, analgesic, muscle relaxant, hepatoprotective, and antiedemal properties. The microalgal biomass can also be used directly in food and pharmaceutical formulations [22, 51].

Chang et al. [52] showed that* Dunaliella* cells contained antibiotic substances. According to these authors, the crude extract of this microalga strongly inhibited the growth of* Staphylococcus aureus*,* Bacillus cereus*,* Bacillus subtilis,* and* Enterobacter aerogenes*. In another study,* Dunaliella* microalga also showed antibacterial activity against various microorganisms of importance to the food industry, including* Escherichia coli*,* Staphylococcus aureus*,* Candida albicans,* and* Aspergillus niger* [49, 53].

Under ideal growing conditions,* Dunaliella* can be stimulated to produce approximately 400 mg of *β*-carotene per square meter of growing area. The cultivation of* Dunaliella* for the production of *β*-carotene has been conducted in several countries, including Australia, Israel, the USA, and China [54–56]. An ingredient of* Dunaliella* with a strong ability to stimulate cell proliferation and improve the energy metabolism of the skin was released by Pentapharm (Basel, Switzerland) [57]. New pilot plants are under development in India, Chile, Mexico, Cuba, Iran, Taiwan, Japan, Spain, and Kuwait [50].

## 3. Cultivation Conditions

The conditions for microalgal cultivation are important factors that influence the metabolism of these microorganisms, thus directing the synthesis of specific compounds of interest. Several researchers have noted the influence of incubation temperature, the pH of the medium, the period of cultivation, as well as salinity, light intensity, and medium constituents, on the synthesis of antimicrobial agents [58].

### 3.1. pH, Temperature, and Luminescence

pH adjustments are the primary measures used to prevent contamination by microorganisms, such as other microalgae species. pH control is also essential for effective absorption of the components of the culture medium because it directly affects the availability of various chemical elements [59]. The reduction of some nutrients in the culture medium can lead the producing of specific biocompounds. The difficulty of consuming a nitrogen source, for example, can lead microalgae to shift your metabolism for lipids or carbohydrates production [60].

Light is an indispensable factor for photosynthesis, causing the cells to reproduce and thereby increasing the cell concentration [61]. The illuminance also influences the biochemical composition of the biomass [62]. The fatty acid content can be reduced with increasing light incidence. This is because lipids are the major components of chloroplasts and the increased light energy demand greater activity of chloroplasts [63]. Studies also show the influence of illuminance on the microalgae antioxidants. According to Madhyastha [64], the application of blue light in the cultivation of the microalga* Spirulina fusiformis* through a phenomenon where the microalgae cells alter the sequence of amino acids with cysteine repeats enhanced the antioxidant capacity.

One of the most important factors for the growth of all living organisms is the temperature. The specific growth rate of the microalgae is directly correlated with the gross rate of CO_2_ fixation/O_2_ production (photosynthesis) and the respiration rate. Photosynthesis and respiration are temperature-dependent, with the respiration rate increasing exponentially with temperature [65]. Temperature has a great influence on the production of biomass, proteins, lipids, and phenolic compounds from microalgae. The optimum temperature for cultivation of microalgae is 35–37°C [66]. In studies conducted by Noaman [58] that were performed to verify which culture conditions stimulated the greatest production of antimicrobial agents by the microalga* Synechococcus leopoliensis, *it was observed that a temperature of 35°C and pH 8 produced a maximum concentration of this bioactive compound.

### 3.2. Bioreactors

Microalgae have attracted much interest for production of bioactive compounds, and in order to grow and tap the potentials of algae, efficient photobioreactors are required. A good number of photobioreactors can be used in production of various algal products [67]. Innovative cultivation systems and modification of biochemical composition of microalgae by simple changes in the growth media and cultivation conditions (nutrients, light intensity, temperature, pH, mixing, etc.) can lead to higher productivity of the targeted products [68].

Bioreactors can be classified as open or closed. Closed photobioreactors have attracted much interest because they allow a better control of the cultivation conditions than open systems. One of the major advantages of open ponds is that they are easier to construct and operate than most closed systems [67].

In open systems, temperature is a main limiting factor, as are variations in solar radiation that lead to low biomass concentrations. However, open systems are the most widely used due to their economic viability. Closed systems are generally used on a pilot scale for investigating problems related to economic viability. Furthermore, the use of closed systems is primarily used for microalgal species that do not grow in a highly selective medium, avoiding contamination of the cultures [69].

Closed bioreactors can provide high productivity, generating greater microalgal biomass per unit time. Other advantages of the use of closed bioreactors compared with open systems include the following: (i) virtually zero losses in connection with evaporation; (ii) a marked reduction of problems related to culture contamination by heterotrophic algae or other microorganisms; (iii) ease of biomass collection procedures due to smaller volumes of culture medium; (iv) greater control of gas exchange between the culture and the atmosphere; (v) a smaller occupied space; (vi) a high surface:volume ratio, which helps to increase the illumination of the system; and (vii) the possibility of obtaining high purity cultures [59].

### 3.3. Nutrients

The metabolism of microalgae can be autotrophic or heterotrophic. The former requires only inorganic compounds, such as CO_2_, salts, and solar energy; the latter is not photosynthetic, requiring an external source of organic compounds for use as a nutrient and energy source. Some photosynthetic species are mixotrophic, having the ability to perform photosynthesis and use exogenous organic sources simultaneously [70].

Microalgae react to changes in their external environment with changes in their intracellular environment. Thus, the manipulation of the culture conditions or the presence or absence of nutrients stimulates the biosynthesis of specific compounds. This fact was first referenced by Richmond [71], who changed the composition of* Chlorella* biomass, particularly in their protein and lipid content, by varying cultivation conditions.

Noaman [58] found that leucine combined with citrate or acetate is the sources of nitrogen and carbon that produced higher concentrations of antimicrobial agents in the microalga* Synechococcus leopoliensis*. Coca et al. [72], studying the cultivation of* Spirulina platensis* in a medium supplemented with vinasse, obtained an increased protein yield compared to the unsupplemented culture medium. Ip and Chen [73], studying the cultivation of* Chlorella zofingiensis* under mixotrophic cultivation conditions, found that low concentrations of nitrate and a high glucose concentration favored the production of astaxanthin in this microalga. Alonso et al. [74], studying the influence of nitrogen concentration in continuous cultivation on lipid concentration in* Phaeodactylum tricornutum*, noted that there was accumulation of saturated and unsaturated fatty acids when the nitrogen source was reduced.

Culture media are chemical preparations that are formulated to contain the nutrients necessary for the microorganisms to multiply and/or survive. The culture media should meet the nutritional needs of the microorganism, assist in process control, and have a reasonably fixed composition [75].

Among different microalgae, variations in the culture medium are mainly related to the amount of necessary nutrients. Even so, nutritional needs are dependent on environmental conditions [59]. Microalgae require macronutrients, such as C, N, O, H, P, Ca, Mg, S, and K, for their growth. The micronutrients that are generally required are Fe, Mn, Cu, Mo, and Co. Additionally, some species require lower concentrations of vitamins in the culture medium [76].

## 4. Advantages of Using Microalgae to Obtain Bioactive Compounds

Microalgae are important sources of bioactive natural substances. Many metabolites isolated from these microorganisms have shown biological activities and potential health benefits [77]. Microalgae accumulate specific secondary metabolites (such as pigments and vitamins) which are high value products that have applications in the cosmetic, food, or pharmaceutical industries [8, 78].

Microalgae live in complex habitats and are subjected to stress and/or extreme conditions, such as changes in salinity, temperature, and nutrients. Thus, these microorganisms must rapidly adapt to new environmental conditions to survive and thus produce a great variety of biologically active secondary metabolites that are not found in other organisms [79]. Some of the advantages of microalgal cultivation may be associated with taxonomic diversity, the diverse chemical composition, the potential for growth in a bioreactor under controlled conditions, and the ability to produce active secondary metabolites in response to the stress induced by extreme exposure conditions [39, 80].

In addition to their natural characteristics, other important aspects related to microalgae are the use of solar energy and carbon dioxide (CO_2_) and a high growth rate which can produce higher yields compared to higher plants. In addition, microalgae can be grown in areas and climates that are unsuitable for agriculture; therefore, microalgae do not compete with arable food production land. The possibility of controlling the production of certain bioactive compounds by manipulation of culture conditions is another advantage of using microalgae [7, 81–83].

The cultivation of microalgae is a major mechanism for reducing excess carbon dioxide (CO_2_) in the atmosphere by biofixation, in which an industrial process uses a CO_2_-rich gas as a carbon source for microalgal growth. This mechanism contributes to a reduction of the greenhouse effect and global warming, further reducing the costs of the carbon source for growth, which is the greatest nutrient requirement for microalgae [13, 84].

The cultivation of microalgae is not seasonal; they are important for food in aquaculture systems and can effectively remove pollutants, such as nitrogen and phosphorus, from wastewater. Moreover, they are the most efficient solar energy biomass converters. Microalgae cultivation via sunlight-dependent systems contributes to sustainable development and natural resource management [13].

The integration of the production process of bioactive metabolites in a biorefinery is a sustainable means of energy production, food production, and the production of products with high added value [7]. The biorefinery concept based on microalgae depends on the efficient use of biomass through fractionation, resulting in several isolated products. This concept encompasses a biorefinery platform, which is capable of offering a wide variety of different products, such as products with applications in pharmaceuticals, medicine, food (protein, fiber), and biofuels [7, 85]. These benefits contribute to the economic viability of microalgal production [7, 8].

## 5. Bioactive Compounds

Bioactive compounds are physiologically active substances with functional properties in the human body. There is great enthusiasm for the development and manufacture of various biocompounds that can potentially be used as functional ingredients, such as carotenoids, phycocyanins, polyphenols, fatty acids, and polyunsaturated compounds [16].

An interest in the production of bioactive compounds from natural sources has recently emerged, driven by a growing number of scientific studies that demonstrate the beneficial effects of these compounds on health [80]. Natural products are important in the search for new pharmacologically active compounds. In general, they play a role in drug discovery for the treatment of human diseases [86]. Many clinically viable and commercially available drugs with antitumor and antiinfective activity originated as natural products.

Microalgae are a natural source of interesting biocompounds. Microalgae are known to produce various therapeutically effective biocompounds that can be obtained from the biomass or released extracellularly into the medium [11]. These microorganisms contain many bioactive compounds, such as proteins, polysaccharides, lipids, vitamins, enzymes, sterols, and other high-value compounds with pharmaceutical and nutritional importance that can be employed for commercial use [13].

### 5.1. Compounds with Antioxidant Function

Oxidative damage caused by reactive oxygen species to lipids, proteins, and nucleic acids can cause many chronic diseases such as heart disease, atherosclerosis, cancer, and aging. Epidemiological studies have demonstrated an inverse association between the intake of fruits and vegetables and mortality from diseases such as cancer. This phenomenon can be attributed to the antioxidant activity of these foods [87].

Microalgal biomass is considered a rich natural source of antioxidants, with potential applications in food, cosmetics, and medicine [87]. Antioxidant compounds, such as dimethylsulfoniopropionate and mycosporine amino acids, were isolated from microalgae and are potent chemical blockers of UV radiation [88]. In addition to these compounds, pigments, lipids, and polysaccharides with antioxidant activity can also be found in microalgal biomass.

Carotenoids and phycocyanins are the pigments most used in scientific research. C-phycocyanin (C-PC) is a blue photosynthetic pigment that belongs to the group of phycobiliproteins found in large quantities in the cyanobacteria, Rhodophyta, and Cryptophyte [89]. Phycocyanin has applications as a nutrient and natural food colorants and cosmetics. It is usually extracted from the biomass of* Spirulina* [90] and* Porphyridium cruentum* [91] and* Synechococcus* [89].

Among the carotenoid compounds, *β*-carotene and astaxanthin are prominent. These compounds have application in the food and pharmaceutical industries because of their antioxidant properties and pigmentation ability. In microalgal metabolism, they protect photosynthetic tissues against damage caused by light and oxygen [92].* Dunaliella salina* is a microalga recognized as a major biological source of *β*-carotene pigment, producing more than 14% in dry biomass [46].* H. pluvialis* is a source of the pigment astaxanthin, producing 1–8% of astaxanthin as dry biomass [93].

Polysaccharides represent a class of high value-added components with applications in food, cosmetics, fabrics, stabilizers, emulsifiers, and medicine [94]. Microalgal polysaccharides contain sulphate esters, are referred to as sulfated polysaccharides, and possess unique medical applications. The basic mechanism of therapeutic action is based on the stimulation of macrophages and modulation. The biological activity of sulfur polysaccharides is linked to their sugar composition, position, and degree of sulfation [95]. Among the microalgae capable of producing these compounds are* Chlorella vulgaris*,* Scenedesmus quadricauda* [96], and* Porphyridium* sp. [97].

### 5.2. Compounds with Antimicrobial Activity

The importance of discovering new compounds with antimicrobial activity is driven by the development of antibiotic resistance in humans due to constant clinical use of antibiotics. Microalgae are an important source of antibiotics with a broad and efficient antibacterial activity [11]. The antimicrobial activity of these microorganisms is due to the ability to synthesize compounds, such as fatty acids, acrylic acids, halogenated aliphatic compounds, terpenoids, sterols, sulfur-containing heterocyclic compounds, carbohydrates, acetogenins, and phenols [98].

The antimicrobial activity of extracts from microalgae is related to its lipid composition. The antimicrobial action of microalgae is also noteworthy because of the potential to produce compounds such as *α*- and *β*-ionone, *β*-cyclocitral, neophytadiene, and phytol [99]. Microalgae antimicrobial activity against human pathogens, such as* Escherichia coli, Pseudomonas aeruginosa, Staphylococcus aureus,* and* Staphylococcus epidermidis*, has been attributed to *γ*-linolenic acid, eicosapentaenoic acid, hexadecatrienoic acid, docosahexaenoic acid, palmitoleic acid, lauric acid, oleic acid, lactic acid, and arachidonic acid [99, 100].

The mechanism of action of fatty acids affects various structures in microorganisms; however cell membranes are the most impacted. Membrane damage most likely leads to a loss of internal substances from the cells, and the entry of harmful components reduces nutrient absorption, in addition to inhibiting cellular respiration. The ability of fatty acids to interfere with bacterial growth depends on both their chain length and the degree of unsaturation. Fatty acids with more than 10 carbon atoms apparently induce lysis of bacterial protoplasts [99].

Microbial polysaccharides and other biological compounds have antiviral and antimicrobial action. Microalgae produce extracellular sulfated polysaccharide (EPS) with acidic characteristics that has a potential as a therapeutic agent [101]. Highly sulfated antiviral polysaccharides from several species of microalgae consist mainly of xylose, glucose, and galactose. The EPS sulfate groups determine some characteristics of polysaccharides; it has been found that higher sulphate contents induced higher antiviral activities [94, 101]. The inhibitory effect of polysaccharides of microalgal origin is due to viral interactions or positive charges on the cell surface, thereby preventing penetration of the virus into host cells [99].

The cyanobacterium* Spirulina* (*Arthrospira*) can produce sulfated polysaccharides that have already found applications as antiviral agents, both* in vivo* and* in vitro* [102]. Eukaryotic microalgae, such as* Chlorella* sp. and* Dunaliella* sp., produce and secrete polysaccharides at relatively high levels [17]. The antibacterial ability of* Spirulina* has been correlated with their volatile composition, resulting in the identification of 15 elements, which constitutes 96% of total compounds. The major volatile components produced by* Spirulina* consist of heptadecane (40%) and tetradecane (35%) [39].

Some studies have reported that sulfated polysaccharides derived from microalgae inhibit viral infection, such as encephalomyocarditis virus, Herpes simplex virus types 1 and 2 (HSV1, HSV2), human immunodeficiency virus (HIV), hemorrhagic septicemia in salmonid virus, swine fever virus, and varicella virus [99, 103]. Carrageenan is a sulfated polysaccharide that can directly bind to human papillomavirus to inhibit not only the viral adsorption process but also the input and subsequent process of the uncoating of the virus [101].

### 5.3. Compounds with Anti-Inflammatory Action

Inflammation is an immediate reaction to a cell or tissue injury caused by noxious stimuli, such as toxins and pathogens. In this situation, the body recognizes the agents responsible for the attack and attempts to neutralize them as quickly as possible. Inflammation causes redness, swelling, heat, and pain, usually located at the site of infection [104]. Ingestion of anti-inflammatory compounds enhances the body's immune response and helps to prevent disease and aids the healing process. Microalgae produce several anti-inflammatory compounds in their biomass that may exert a protective function in the body when consumed as food or used as pharmaceuticals and cosmetics.

Because of its anti-inflammatory capabilities, microalgal biomass is being considered for applications in tissue engineering for the development of scaffolds, for use in reconstitution of organs and tissues [105, 106]. This is an important application for humans, especially in patients with burns in which the skin was completely lost [107]. Among the most important microalgal compounds with such properties are long-chain polyunsaturated fatty acids (PUFAs) [108, 109], sulfurized polysaccharides [110], and pigments [111].

Many microalgal polysaccharides possess the ability to modulate the immune system through the activation of macrophage functions and the induction of reactive oxygen species (ROS), nitric oxide (NO), and various other types of cytokines/chemokines [112]. Macrophages are able to regulate several innate responses and secrete cytokines and chemocytokines that serve as signals for immune and inflammatory molecular reactions [113]. Sulfur polysaccharides with anti-inflammatory activity can be applied in skin treatments inhibiting the migration and adhesion of polymorphonuclear leukocytes [110]. Guzmán et al. [114] studied the anti-inflammatory capacity of the microalga* Chlorella stigmatophora* and* Phaeodactylum tricomutum* and concluded that both microalgae showed positive responses in the test of paw edema by carrageenan.

The PUFAs, especially *ω*3 and *ω*6 as eicosapentaenoic (EPA), docosahexaenoic (DHA), and arachidonic (AA) acids, have been applied in the treatment of chronic inflammation such as rheumatism and skin diseases [108]. Ryckebosch et al. [115] evaluated the nutritional value of the total lipids extracted from different PUFAs produced by microalgae. In this study, the microalgae* Isochrysis*,* Nannochloropsis, Phaeodactylum, Pavlova,* and* Thalassiosira *produced *ω*3 PUFA as an alternative to fish oil in food.

Among the pigments with anti-inflammatory activity, fucoxanthin carotenoid found in diatoms [116, 117] is capable of stimulating apoptosis in human cancer cells [118]. A phycocyanin, found in cyanobacteria, has an anti-inflammatory activity that occurs through the inhibition of histamine release [111, 119].

### 5.4. Compounds with Potentiality over Degenerative Diseases

In humans, the oxidation reactions driven by reactive oxygen species (ROS) can lead to irreversible damage to cellular components, including lipids, proteins, and DNA degradation and/or mutation. Consequently, this damage can lead to several syndromes, such as cardiovascular disease, some cancers, and the degenerative diseases of aging [120].

Chronic age-related diseases involve oxidative stress and inflammation and their consequences. Chronic inflammation plays a significant role in the mediation of neurodegenerative diseases such as Parkinson's disease, Alzheimer's disease, multiple sclerosis, acquired immunodeficiency syndrome (AIDS), and dementia complex [77].

Natural pigments derived from microalgae (NPs) have neuroprotective properties, being valuable sources as functional ingredients in foods and pharmaceutical products that show efficient action in the treatment and/or prevention of neurodegenerative diseases. Vitamin E has preventive effects for many diseases, such as atherosclerosis and heart disease, as well as neurodegenerative diseases, such as multiple sclerosis [77].

Carotenoids have great potential benefits to human health, including the treatment of degenerative diseases, such as macular degeneration and cataract development. These compounds act as antioxidants, reducing oxidative damage by ROS. Studies indicated that increased intake of phenols decreased the occurrence of degenerative diseases. Phenolic compounds from microalgae with the potential to fight free radicals have been reported [121].

*Dunaliella salina* is a natural source of *β*-carotene, which produced a reduced risk of cancer and degenerative diseases in humans. Lutein is effective against various diseases, including cataracts and macular degeneration, and in the early stages of atherosclerosis. Extracts of* Chlorella* sp. containing *β*-carotene and lutein significantly prevented the cognitive disability that accompanies Alzheimer's disease in rats. It was also reported that lutein extracted from* Chlorella* reduced the incidence of cancer. Likewise, carotenoids extracted from* Chlorella ellipsoidea* and* Chlorella vulgaris* inhibited the growth of colon cancer [122]. A lycopene extracted from the microalgae* Chlorella marina* significantly reduced the proliferation of prostate cancer in mice [123]. This compound also reduced total cholesterol and low-density lipoprotein (LDL) levels [123] and improved rheumatoid arthritis [124].

Low plasma levels of lutein have also been associated with an increased tendency of myocardial infarction, whereas high intake of lutein was related to a decreased risk of stroke. In addition, high levels of carotenoids with provitamin A activity, including *α*-carotene, *β*-carotene, and *β*-cryptoxanthin, have been associated with reduction in the risk of angina pectoris. Macular degeneration, the leading cause of irreversible vision loss, has also been associated with very low consumption of lutein and zeaxanthin [125].

Scientific findings indicate astaxanthin for multimodal intervention for many forms of degenerative diseases, including cardiovascular diseases, cancer, metabolic syndrome, cognitive impairment, age-related immune dysfunction, stomach and ocular diseases (macular degeneration, cataract, glaucoma, diabetic retinopathy, and retinitis pigmentosa), and skin damage [126]. High levels of lycopene in plasma and tissues were inversely related to coronary heart disease, myocardial infarction, and the risk of atherosclerosis [125].

### 5.5. Compounds with Health Promoting Function

The importance of microalgae as sources of functional ingredients has been recognized because of their beneficial health effects. Natural pigments are valuable sources of bioactive compounds. These pigments have various beneficial biological activities such as antioxidant, anticancer, anti-inflammatory, antiobesity, antiangiogenic, and neuroprotective action and are indicated for the treatment or prevention of several chronic diseases [77].

The antioxidant potential of carotenoid pigments and their ability to prevent cancer, aging, atherosclerosis, coronary heart disease, and degenerative diseases have been described. *β*-Carotene has higher provitamin A activity, which is essential for vision and the correct functioning of the immune system. Astaxanthin is linked to many health benefits such as protection against lipid peroxidation, age-related macular degeneration, reduced atherosclerosis, and an increased immune response [102].

Fucoxanthin is considered as a promising dietary and weight loss supplement and for the treatment of obesity. Clinical studies by Abidov et al. [127] demonstrated the effect of “xanthigen,” a fucoxanthin based antiobesity supplement. Furthermore, fucoxanthin may be useful for the prevention of bone diseases such as osteoporosis and rheumatoid arthritis. It has also been reported to be effective for the therapeutic treatment of diabetic diseases, suppressing insulin and hyperglycemia [77].

Microalgae proteins are of great interest as a source of bioactive peptides due to their therapeutic potential in the treatment of various diseases [7]. Proteins, peptides, and amino acids have functions that contribute to health benefits. These compounds can include growth factors, hormones, and immunomodulators and can help to replace damaged tissues, in addition to providing nutritional benefits. Microalgae, such as* Chlorella* and* Spirulina* (*Arthrospira*), may be used as nutraceuticals or included in functional foods to prevent diseases and damage to cells and tissues due to their rich protein content and amino acid profile [102].

The antimicrobial action of certain enzymes (e.g., lysozyme) and immunoglobulins has been reported and recommended for people with different diseases (e.g., Crohn's disease) due to the existence of formulations with peptides and free amino acids. Studies of the health effects of lysozyme have been reported in the microalgae* Spirulina platensis *[128],* Chlorella* [129], and* Dunaliella salina* [130].* Spirulina *(*Arthrospira*) and* Chlorella* biomass pills are marketed, as is “Hawaiian Spirulina Pacifica” (http://spirulina.greennutritionals.com.au/). Other proteins can also increase the production of cholecystokinin involved in appetite suppression and the reduction of LDL-cholesterol. Protein peptides from* Chlorella* have a potential as dietary supplements for the prevention of oxidative stress-related diseases, such as atherosclerosis, coronary heart disease, and cancer [39].

The essential fatty acids, *ω*-3 and *ω*-6 in particular, are important for the integrity of tissues. *γ*-Linolenic acid has therapeutic applications in cosmetics, to revitalize the skin and thus slow aging. Linoleic and linolenic acids are essential nutrients for the immune system and other related tissue regeneration processes. Linoleic acid is also used for the treatment of hyperplasia of the skin [102].

The most studied microalgal lipid compounds are the polyunsaturated fatty acids (PUFAs) docosahexaenoic acid (*ω*-3 C22:6) (DHA), eicosapentaenoic acid (C20 *ω*-3:5) (EPA), and arachidonic acid (*ω*-6 C20:4) (ARA). Studies have shown that dietary *ω*-3 PUFAs have a protective effect against atherosclerotic heart disease [131]. DHA and EPA showed the ability to reduce problems associated with strokes and arthritis, besides reducing hypertension, lipid content (a decrease in triglycerides and an increase of HDL) and acting as anti-inflammatory agents. DHA is also important in the development and function of the nervous system. Furthermore, ARA and EPA are platelet aggregators, vasoconstrictors, and vasodilators and have antiaggregative action on the endothelium, as well as chemostatic activity in neutrophils [102].

Other lipid compounds with interesting bioactive properties are the microalgal sterols. Phytosterols have demonstrated reduction of total cholesterol (LDL) in humans by inhibiting its absorption from the intestine [50]. Polysaccharides can be considered as dietary fibers associated with different physiological effects. Insoluble fiber (cellulose, hemicellulose, and lignin) mainly promotes the movement of material through the digestive system, thereby improving laxation and increasing satiety. They can also be considered as prebiotics because they promote the growth of gut microflora, including probiotic species. Soluble fiber (oligosaccharides, pectins, and *β*-glucans) may reduce cholesterol and regulate blood glucose [7, 132].

## 6. Conclusion

The proven ability of microalgae to produce bioactive compounds places these microorganisms in the biotechnological spotlight for applications in various areas of study, especially in the life sciences. The production of microalgal metabolites, which stimulate defense mechanisms in the human body, has spurred intense study of the application of microalgal biomass in various foods and pharmacological and medical products. There is obviously a need for further study of the identified compounds and their activities in the treatment and prevention of various diseases, in addition to an ongoing search for other, as yet undetected, metabolites.

## Figures and Tables

**Table 1:** Principal bioactive compounds extracted from microalgae.

Microalgae	Bioactive compounds	Reference

*Spirulina *sp.	Polysaccharides	[133]
*Spirulina platensis *	Phycocyanin, C-phycocyanin, Phenolic acids, tocopherols (vitamin E), neophytadiene, phytol, PUFAs (*n*-3) fatty acids, oleic acid, linolenic acid, palmitoleic acid	[7, 39, 81]
*Spirulina fusiformis *	Diacylglycerols	[81]
*Haematococcus pluvialis *	Astaxanthin, lutein, zeaxanthin, canthaxanthin, lutein, *β*-carotene, oleic acid	[8, 39, 81]
*Chlorella *sp.	Carotenoids, sulfated polysaccharides, sterols, PUFAs (*n*-3) fatty acids	[7]
*Chlorella vulgaris *	Canthaxanthin, astaxanthin, peptide, oleic acid	[13, 39, 133]
*Chlorella minutissima *	Eicosapentaenoic acid (EPA)	[81]
*Chlorella ellipsoidea *	Zeaxanthin, violaxanthin	[133]
*Dunaliella salina *	*trans*-Betacarotene, *cis*-betacarotene, *β*-carotene, oleic acid, linolenic acid, palmitic acid	[12, 39, 81]
*Dunaliella *	Diacylglycerols	[81]
*Botryococcus braunii *	Linear alkadienes (C25, C27, C29, and C31), triene (C29)	[12]
*Chlorella zofingiensis *	Astaxanthin	[8]
*Chlorella protothecoides *	Lutein, zeaxanthin, canthaxanthin	[8, 102]
*Chlorella pyrenoidosa *	Lutein, sulfated polysaccharide	[39]
*Nostoc linckia * and *Nostoc spongiaeforme *	Borophycin	[81]
*Nostoc *sp.	Cryptophycin	[81]

**Table 2:** Bioactive compounds extracted from *Spirulina* genus.

Microalga	Bioactive compound	Concentration (%, w/w)	Reference

*Spirulina fusiformis *	C-phycocyanin	46.0	[8]
*Spirulina platensis *	C-phycocyanin	9.6	[8]
*Spirulina platensis *	Allophycocyanin	9.5	[8]
*Spirulina *sp.	C-phycocyanin	17.5	[8]
*Spirulina *sp.	Allophycocyanin	20.0	[8]
*Spirulina platensis *	Phenolic	0.71	[134]
*Spirulina platensis *	Terpenoids	0.14	[134]
*Spirulina platensis *	Alkaloids	3.02	[134]
*Spirulina maxima *	Phenolic	1.29	[121]
*Spirulina maxima *	Flavonoids	0.46	[121]

**Table 3:** Bioactive compounds extracted from the *Nostoc* genus.

Microalga	Bioactive compound	Concentration (%)	Reference

*Nostoc *sp.	Phycocyanin	20.0 (p/p)	[8]
*Nostoc muscorum *	Phenolic	0.61 (p/p)	[134]
*Nostoc muscorum *	Terpenoids	0.10 (p/p)	[134]
*Nostoc muscorum *	Alkaloids	2.30 (p/p)	[134]
*Nostoc muscorum *	Phycobilins	0.0229 (p/v)	[134]
*Nostoc humifusum *	Phenolic	0.34 (p/p)	[134]
*Nostoc humifusum *	Terpenoids	0.10 (p/p)	[134]
*Nostoc humifusum *	Alkaloids	1.65 (p/p)	[134]
*Nostoc humifusum *	Phycobilins	0.0031 (p/v)	[134]

**Table 4:** Bioactive compounds extracted from the microalgae of the *Chlorella* genus.

Microalga	Bioactive compound	Concentration (%, w/w)	Reference

*Chlorella protothecoides *	Lutein	4.60	[8]
*Chlorella zofingiensis *	Astaxanthin	1.50	[8]
*Chlorella vulgaris *	Phenolic	0.20	[134]
*Chlorella vulgaris *	Terpenoids	0.09	[134]
*Chlorella vulgaris *	Alkaloids	2.45	[134]
*Chlorella minutissima *	Phytol	2.70	[135]
*Chlorella minutissima *	Phenol	1.81	[135]

**Table 5:** Bioactive compounds extracted from the microalgae of the *Dunaliella *genus.

Microalga	Bioactive compound	Concentration (%, w/w)	Reference

*Dunaliella salina *	*β*-Carotene	12%	[8]
*Dunaliella salina *	All-*trans*-*β*-carotene	13.8%	[136]
*Dunaliella salina *	All-*trans*-zeaxanthin	1.1%	[136]
*Dunaliella salina *	All-*trans*-lutein	0.66%	[136]
*Dunaliella tertiolecta *	Sterols	1.3%	[50]
*Dunaliella salina *	Sterols	0.89%	[50]
